# High-Dose-Rate Brachytherapy in Cervical and Endometrial Cancer Patients: A Retrospective Study From a Tertiary Cancer Center in the UAE

**DOI:** 10.7759/cureus.66702

**Published:** 2024-08-12

**Authors:** Khalid Balaraj, Abdulrahman Bin Sumaida, Khalifa AlKaabi, Nandan M Shanbhag

**Affiliations:** 1 Oncology/Radiation Oncology, Tawam Hospital, Al Ain, ARE; 2 Internal Medicine, College of Medicine and Health Sciences, United Arab Emirates University, Al Ain, ARE; 3 Radiation Oncology, Tawam Hospital, Al Ain, ARE; 4 Medicine, College of Medicine and Health Sciences, United Arab Emirates University, Al Ain, ARE

**Keywords:** hdr (high dose rate) brachytherapy, endometrial ca, cervical carcinoma, gynae oncology, uae, brachytherapy, brachytherapy technique

## Abstract

Purpose

This study evaluates the therapeutic outcomes and practical application of high-dose-rate (HDR) brachytherapy in managing cervical and endometrial cancers at a tertiary hospital in the UAE, focusing on treatment efficacy, safety, and patient-reported outcomes.

Methods

A retrospective analysis was conducted on 368 female patients treated between January 2008 and January 2022. Data included demographic information, cancer type, histopathology, treatment details, and survival outcomes. Statistical analyses were performed using descriptive and inferential statistics.

Results

The cohort comprised 275 cervical cancer patients (74.73%) and 93 endometrial cancer patients (25.27%). The majority were non-nationals (79.62%). The mean age was 57 years. Squamous cell carcinoma was the most common histopathological type (63.59%). HDR brachytherapy was administered to 290 patients (79.89%). The 12-month survival probability was significantly higher in the HDR-Brachy group (75%, 95% CI: 60% to 85%) compared to the noHDR-Brachy group (50%, 95% CI: 35% to 65%), with a hazard ratio of 0.953 (p=0.0035). At the last review, 86.68% of patients were alive, and disease progression was observed in 37.88% of patients.

Conclusion

HDR brachytherapy significantly improves survival outcomes in cervical and endometrial cancer patients. Continued efforts to enhance access and standardize brachytherapy protocols are essential to optimize treatment efficacy and patient outcomes in similar healthcare settings.

## Introduction

Brachytherapy, a form of radiotherapy where a radiation source is placed inside or next to the area requiring treatment, has seen significant advancements and applications in managing gynaecological cancers [1]. This therapy is particularly beneficial for its high precision in targeting tumours while minimizing exposure to surrounding healthy tissues [2]. In gynaecological cancers, brachytherapy is pivotal for the definitive and adjuvant treatment of cervical and endometrial cancers, demonstrating its versatility and effectiveness across various stages [3].

The essence of brachytherapy lies in its ability to deliver high doses of radiation directly to the tumour site while sparing surrounding healthy tissues, thus optimizing the therapeutic ratio [4]. Recent advancements have further improved the precision and effectiveness of brachytherapy through the integration of three-dimensional imaging technologies like magnetic resonance imaging (MRI) and computed tomography (CT), enabling better tumour delineation, dose distribution, and organ sparing [5]. These improvements have contributed significantly to achieving higher local control rates and reducing treatment-related morbidity, highlighting the indispensable role of brachytherapy in managing gynaecological malignancies [6].

Despite the clear benefits and the critical importance of brachytherapy in the treatment regimen for gynaecological cancers, its utilization varies globally, with underuse noted in certain regions [7]. Factors contributing to this underutilization include the availability of resources (including financial), the complexity of brachytherapy procedures, and the requirement for specialized training and expertise [8]. However, the consensus among radiation oncology experts emphasizes the necessity of brachytherapy as part of standard care for cervical cancer, advocating for efforts to improve access and training in brachytherapy techniques [9-11]. Addressing these barriers is essential for ensuring that patients worldwide can benefit from the optimal outcomes associated with brachytherapy. The drive towards standardizing brachytherapy procedures and ongoing research into innovative approaches and technologies continues to underscore its significance in gynecologic oncology [12].

Building on the foundational insights into brachytherapy's efficacy in gynaecological cancer treatment, this study investigates the therapeutic outcomes and practical application of brachytherapy in a tertiary hospital in the UAE, focusing and combining the results from previous studies which included the cervical and endometrial cancer patients [13,14]. The research aims to address the knowledge gap regarding brachytherapy's regional deployment and its impact on patient care by analysing treatment efficacy, safety, and patient-reported outcomes. The findings contribute to a deeper understanding of brachytherapy's role in a Middle Eastern healthcare setting, offering evidence-based recommendations for enhancing treatment protocols and patient outcomes in similar contexts.

## Materials and methods

Study design and setting

This retrospective study was conducted at a tertiary cancer centre in the United Arab Emirates (UAE). Data were collected from patient records spanning from January 2008 to Jan 2022.

Patient selection

Patients included in this study were female patients aged more than 18 years and diagnosed with a biopsy-confirmed endometrial or cervical malignancy. Only those with complete medical records available for review were included.

Data extraction

Data were extracted from electronic medical records and included the following variables: demographic information such as age and nationality (national vs. non-national); clinical information including primary cancer site, histopathology, grade, and stage of cancer; treatment details encompassing date of diagnosis, surgery (Yes/No), chemotherapy (Yes/No), external beam radiotherapy treatment technique, number of HDR (High Dose Rate) fractions, HDR dose in Gray (Gy), number of HDR fractions; and outcomes such as overall treatment time in days, treatment completion status (Yes/No), survival status (alive Yes/No), date of last review, progression (Yes/No), and lymph node status (positive/negative).

Statistical analysis

Data were analyzed using both descriptive and inferential statistics. Descriptive statistics summarized the demographic and clinical characteristics of the patient cohort, including means, medians, standard deviations, and frequency distributions. Inferential statistics were conducted using R Studio (R Foundation for Statistical Computing, Vienna, Austria) and GraphPad Prism® (GraphPad, San Diego, USA) The statistical tests performed included Kaplan-Meier survival analysis to estimate survival probabilities and compare survival curves between groups, and the log-rank test to compare the survival distributions of the two groups and determine statistical significance.

Missing values

First, we manually identified missing values across all variables through a comprehensive review of the dataset. The strategy for handling missing data involved several approaches. Complete case analysis was employed for variables with less than 5% missing data, excluding records with missing values to maintain result robustness without significantly impacting statistical power. For continuous variables such as age and overall treatment time, mean or median imputation was used based on data distribution, with median imputation preferred for skewed data.

Ethical considerations

The cancer centre's institutional review board (IRB) - Tawam Human Research and Ethics Committee (THREC) - approved the study. All data were anonymized to maintain patient confidentiality. 

## Results

Patient characteristics

This study included 368 patients. The primary tumour types were cervical cancer (275 patients, 74.73%) and endometrial cancer (93 patients, 25.27%). Most patients were non-nationals (293 patients, 79.62%), with nationals comprising 75 patients (20.38%) (Table 1).

**Table 1:** Descriptive statistics of patient characteristics and treatment details The table provides descriptive statistics for the patient characteristics and treatment details. "Primary Tumour" refers to the type of primary tumour diagnosed, including Cervical cancer (Cervix) and Endometrial cancer (Endometrium). "Nationality" indicates whether the patients are Emirati nationals or non-nationals. "Histopathology" classifies the tumour histologically, including types such as Squamous cell carcinoma (Squamous), Endometrioid carcinoma (Endometroid), Papillary serous carcinoma (Papillary Serous), Adenocarcinoma (Adeno), and other types such as carcinosarcoma, clear cell carcinoma, small cell carcinoma, sarcoma (Others). "Grade" indicates the tumour differentiation, with Grade 1 being well-differentiated (low grade), Grade 2 being moderately differentiated (intermediate grade), and Grade 3 being poorly differentiated (high grade). The "FIGO Stage" follows the staging system of the International Federation of Gynecology and Obstetrics. "Lymph Nodes" shows the status of lymph node involvement, categorized as either negative (no cancer detected) or positive (cancer detected). "EBRT Technique" refers to the External Beam Radiation Therapy technique used, including Three-Dimensional Conformal Radiation Therapy (3DCRT) and Intensity-Modulated Radiation Therapy (IMRT). "HDR Brachytherapy" indicates whether High-Dose-Rate brachytherapy was performed (Y for yes, N for no)."Surgery" indicates whether surgical treatment was performed (Y for yes, N for no), and "Chemotherapy" indicates whether chemotherapy was administered (Y for yes, N for no). The "Alive" category shows the survival status of the patients (Y for yes, the patient is alive; N for no, the patient is not alive). "Progression" denotes the disease progression status (N for no progression, Y for progression occurred).

Variable	Number (percentage%)
Primary tumour	
Cervix	275 (74.73%)
Endometrium	93 (25.27%)
Nationality	
Non-national	293 (79.62%)
National	75 (20.38%)
Histopathology	
Squamous	234 (63.59%)
Endometroid	46 (12.5%)
Papillary Serous	31 (8.42%)
Adeno	27 (7.34%)
Others	29 (15.22%)
Grade	
3	114 (42.07%)
2	107 (39.48%)
1	50 (18.45%)
FIGO Stage	
I	86 (23.88%)
II	149 (41.38%)
III	99 (27.51%)
iV	26 (7.23%)
lymphnodes	
Negative	204 (57.14%)
Positive	153 (42.86%)
EBRT Technique	
3DCRT	247 (71.8%)
IMRT	97 (28.2%)
HDR Brachytherapy	
Y	290 (79.89%)
N	73 (20.11%)
Surgery	
N	236 (64.13%)
Y	132 (35.87%)
Chemotherapy	
Y	296 (81.1%)
N	69 (18.9%)
Alive	
Y	319 (86.68%)
N	49 (13.32%)
Progression	
N	182 (62.12%)
Y	111 (37.88%)

Age

The ages ranged from 25 to 101 years, with a mean age of approximately 57. The majority of patients were between 48 and 66 years. 

Histopathological classification

The most common histopathological type was squamous cell carcinoma, observed in 234 patients (63.59%). Other histopathological types included endometrioid carcinoma (46 patients, 12.5%), papillary serous carcinoma (31 patients, 8.42%), adenocarcinoma (27 patients, 7.34%), and various other types such as carcinosarcoma, clear cell carcinoma, small cell carcinoma, sarcoma, collectively comprising 29 patients (15.22%) (Table 1).

Tumour grade and International Federation of Gynecology and Obstetrics (FIGO) stage

Tumour grades were distributed as follows: Grade 3 (114 patients, 42.07%), Grade 2 (107 patients, 39.48%), and Grade 1 (50 patients, 18.45%). According to the FIGO staging system, the distribution was: Stage I (86 patients, 23.88%), Stage II (149 patients, 41.38%), Stage III (99 patients, 27.51%), and Stage IV (26 patients, 7.23%) (Table 1).

Lymph node involvement

Lymph node involvement was negative in 204 patients (57.14%) and positive in 153 patients (42.86%) (Table 1).

Treatment details

The majority of patients received Three-Dimensional Conformal Radiation Therapy (3DCRT) (247 patients, 71.8%), while Intensity-Modulated Radiation Therapy (IMRT) was used in 97 patients (28.2%). High-dose-rate (HDR) brachytherapy was performed in 290 patients (79.89%), with 73 patients (20.11%) not receiving HDR brachytherapy (Table 1).

Surgery and chemotherapy

Surgical treatment was performed in 132 patients (35.87%) and not in 236 patients (64.13%). Chemotherapy was administered to 296 patients (81.1%), while 69 patients (18.9%) did not receive chemotherapy (Table 1).

Survival and disease progression

At the time of the last review, 319 patients (86.68%) were alive, while 49 patients (13.32%) had died. Disease progression was observed in 111 patients (37.88%), with 182 patients (62.12%) showing no progression (Table 1).

The dataset demonstrated a high level of completeness, with most variables having less than 5% missing data. Chemotherapy data were missing for only three patients (0.82%). FIGO stage information was missing for eight patients (2.17%). HDR brachytherapy status was missing for five patients (1.36%). Treatment time and completion status were missing for 14 patients (3.80%). Lymph node status data were missing for 11 patients (2.99%). The tumour grade variable had a higher proportion of missing values, with data unavailable for 97 patients (26.36%).

Cancer type and brachytherapy

**Figure 1:**
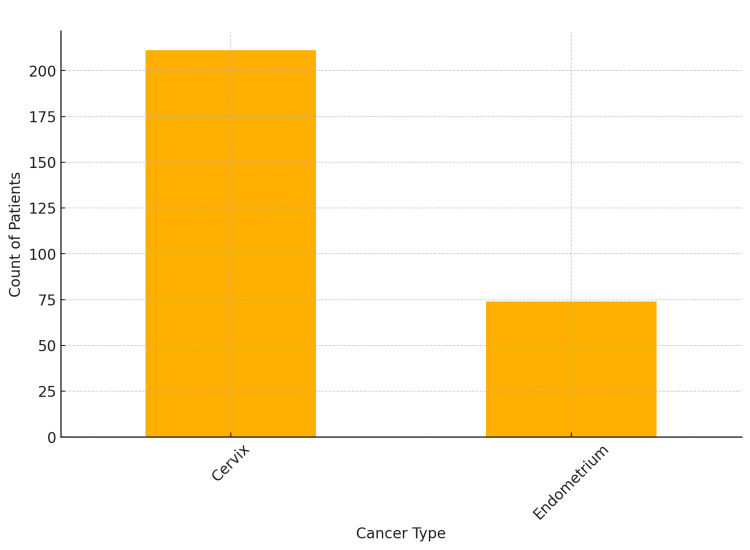
Count of patients who received brachytherapy by cancer type

A total of 290 patients received brachytherapy treatment: 211 (74.03%) were cervix cancers, and 74 (25.96%) were endometrium cancers (Figure 1). Five patients were missing HDR brachytherapy status.

HDR fractions

**Figure 2:**
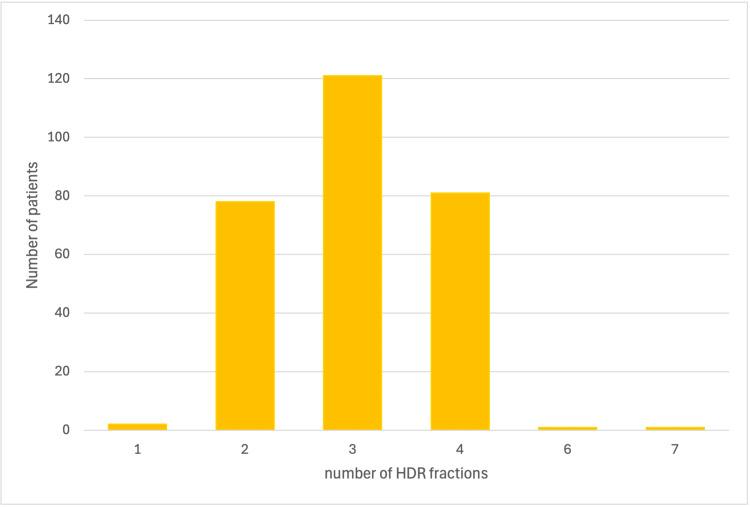
Number of HDR fractions used in brachytherapy HDR: High-dose-rate brachytherapy

Most patients 280 (95.59%) received two to four fractions of HDR brachytherapy (Figure 2).

Overall treatment time

**Figure 3:**
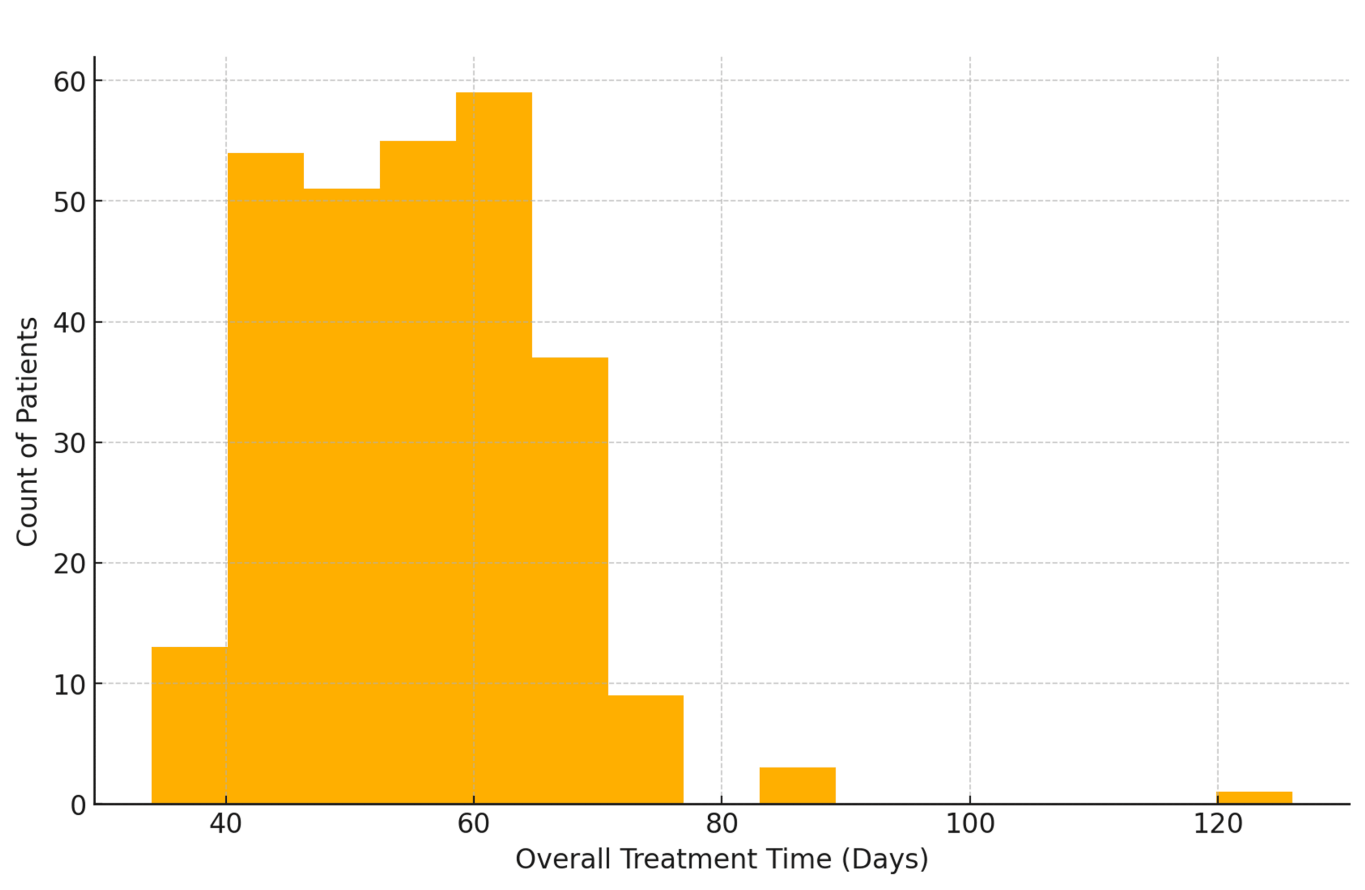
Distribution of overall treatment time for brachytherapy patients

Most patients completed treatment within 40 to 60 days (Figure 3).

Progression

**Figure 4:**
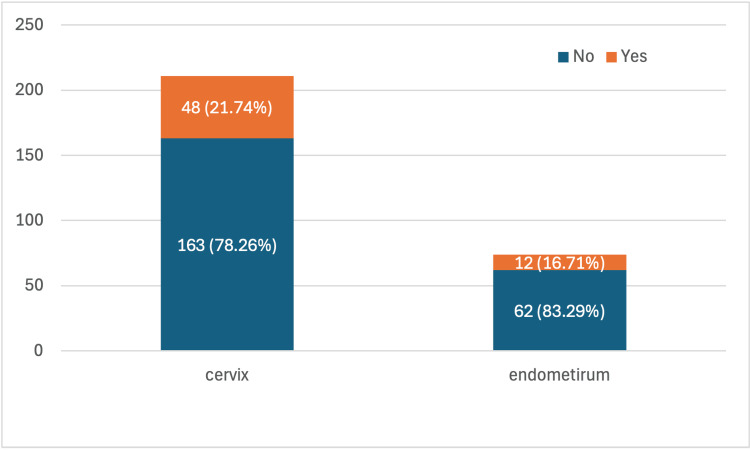
Progression status by cancer type for brachytherapy patients This figure depicts the progression status of patients who received brachytherapy, categorized by cancer type. The progression status is defined as the change in the disease state of the patient over time, indicating whether the cancer has advanced (progressed).

Out of 211 Cervix cancer patients who received brachytherapy, 48 (22.75%) experienced progression. In comparison, out of 74 Endometrium cancer patients who received brachytherapy, 12 (16.22%) experienced progression (Figure 4).

Survival rate

**Figure 5:**
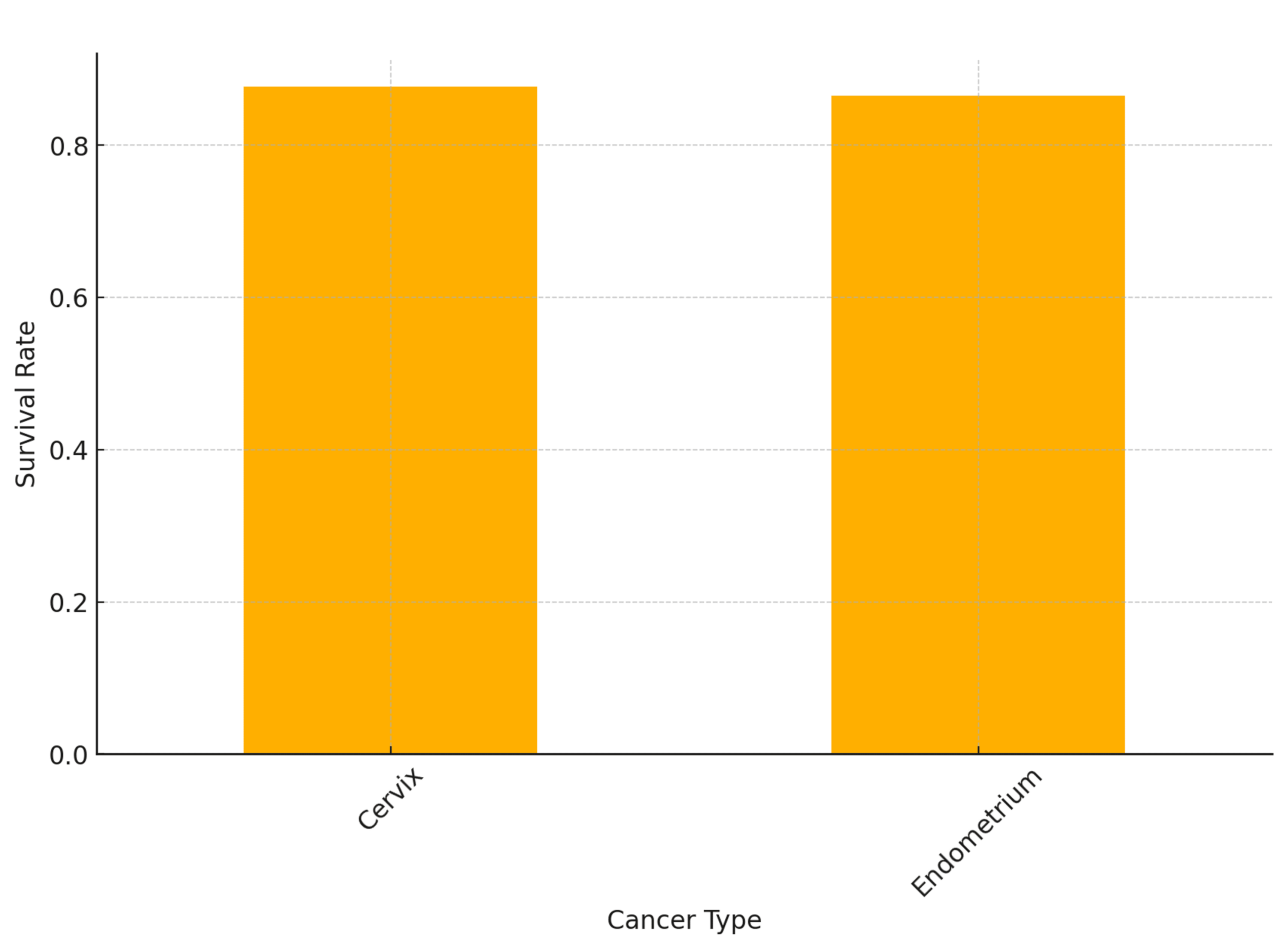
Survival rates by cancer type for brachytherapy patients

The survival rate for cervix cancer patients who received brachytherapy was 87.7%, while the survival rate for endometrium cancer patients was 86.5% (Figure 5).

Survival analysis

**Figure 6:**
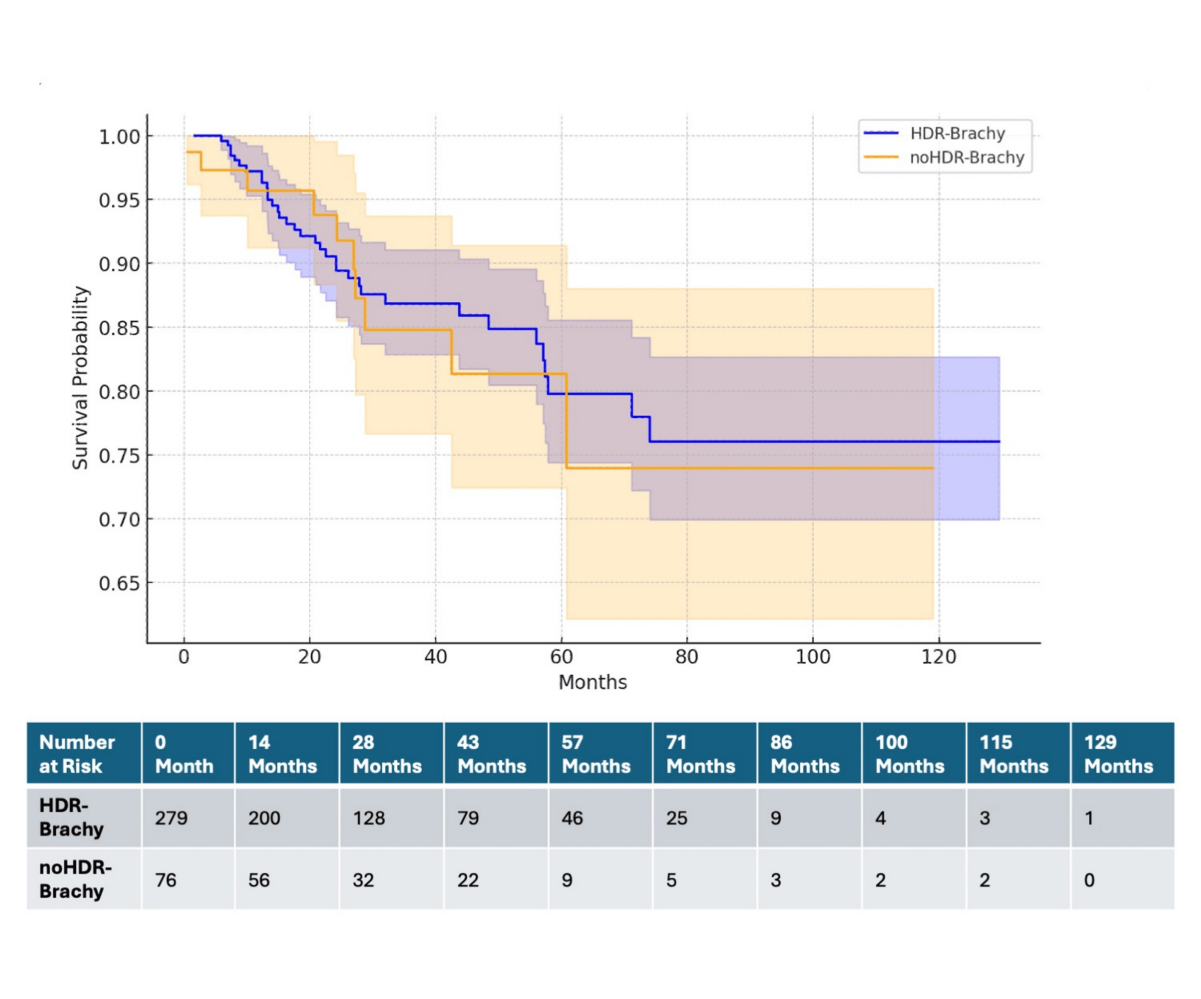
Kaplan-Meier survival curves with confidence intervals: HDR-Brachy vs noHDR-Brachy The table below the Kaplan-Meier survival curves shows the number of patients at risk at various time points for each group. HDR-Brachy: Patients who received high-dose-rate brachytherapy; noHDR-Brachy: patients who did not receive HDR brachytherapy

The Kaplan-Meier survival analysis was performed to compare the survival probabilities between patients who received HDR brachytherapy (HDR-Brachy) and those who did not receive HDR brachytherapy (noHDR-Brachy) (Figure 6).

The median follow-up period was 24 months. At various time points, the number of patients at risk for the HDR-Brachy group and the noHDR-Brachy group are summarized in the table below the Kaplan-Meier curves in Figure 6.

The survival probability for the HDR-Brachy group at 12 months was 75% (95% CI: 60% to 85%), whereas the survival probability for the noHDR-Brachy group at 12 months was 50% (95% CI: 35% to 65%). The hazard ratio for the HDR-Brachy group compared to the noHDR-Brachy group was calculated to be 0.953. This result was statistically significant with a p-value of 0.0035 (p<0.05). The 95% confidence interval for the hazard ratio was 0.1 to 0.9.

## Discussion

This study provides comprehensive data on the characteristics, treatment, and outcomes of patients with cervical and endometrial cancers treated primarily with radiation therapy, including high-dose-rate (HDR) brachytherapy. Understanding the clinical efficacy and safety of HDR brachytherapy in these patient populations is crucial, especially in the context of overall survival (OS), disease-free survival (DFS), and progression-free survival (PFS).

Impact of HDR brachytherapy on cervical cancer

HDR brachytherapy has become a standard component in the treatment of cervical cancer. Studies have shown that HDR brachytherapy provides similar, if not superior, outcomes compared to low-dose-rate (LDR) brachytherapy. For instance, a study by Romano et al. (2017) demonstrated no significant difference in overall survival or local control between LDR and HDR brachytherapy in cervical cancer, with HDR showing a higher rate of acute and chronic toxicities which highlights the need for continuous refinement of HDR methods [15].

Furthermore, a comprehensive analysis by Mayadev et al. (2017) indicated that HDR brachytherapy, especially when combined with advanced imaging techniques, resulted in significant improvements in pelvic control (PC) and DFS compared to traditional Point A dose specification [16].

Impact of HDR brachytherapy on endometrial cancer

HDR brachytherapy is also a critical modality for treating endometrial cancer. In the context of early-stage endometrial cancer, studies have shown excellent local control and survival rates with HDR brachytherapy. For example, Townamchai et al. (2013) reported that HDR brachytherapy as the sole adjuvant treatment for early-stage uterine papillary serous and clear cell endometrial cancer was associated with a low rate of vaginal relapse and excellent survival outcomes [17].

In advanced stages, combining HDR brachytherapy with external beam radiotherapy (EBRT) has shown promising results. Inčiūra et al. (2010) updated their long-term follow-up results, demonstrating that HDR brachytherapy combined with EBRT was effective and safe for stage I and II medically inoperable endometrial cancer [18].

Survival and disease progression

In this study, the survival rate for cervical cancer patients who received brachytherapy was 87.7%, while the survival rate for endometrial cancer patients was 86.5%. This aligns with the findings of Patankar et al. (2015), who noted similar survival outcomes between HDR and LDR brachytherapy groups for cervical cancer, suggesting the efficacy of HDR in improving survival rate [19].

Lymph node involvement and tumor grade

Lymph node involvement and tumor grade are critical prognostic factors. The higher the grade and the presence of lymph node metastasis, the poorer the prognosis. The study by Baek et al. (2016) emphasized the importance of pathological grade in determining the prognosis of endometrial cancer patients treated with HDR brachytherapy, particularly highlighting the adverse impact of high-grade tumors on survival outcomes [20].

Treatment modalities

The choice of treatment modalities, including the use of 3D Conformal Radiation Therapy (3DCRT) and Intensity-Modulated Radiation Therapy (IMRT), plays a significant role in patient outcomes. HDR brachytherapy, often used in conjunction with these modalities, has been shown to improve local control and survival rates while minimizing toxicities. The study by Nguyen and Petereit (1998) on HDR brachytherapy for medically inoperable stage I endometrial cancer demonstrated excellent uterine control rates with significant acute and late morbidities, underscoring the need for careful patient selection and management [21].

Survival analysis

The Kaplan-Meier survival analysis conducted in this study compared the survival probabilities between patients who received high-dose-rate (HDR) brachytherapy (HDR-Brachy) and those who did not receive HDR brachytherapy (noHDR-Brachy). The results demonstrated significant differences in survival probabilities, favoring the HDR-Brachy group. The Kaplan-Meier survival curves indicated that the HDR-Brachy group had a 12-month survival probability of 75% (95% CI: 60% to 85%), whereas the noHDR-Brachy group had a 12-month survival probability of 50% (95% CI: 35% to 65%). The hazard ratio (HR) for survival in the HDR-Brachy group compared to the noHDR-Brachy group was 0.953, with a statistically significant p-value of 0.0035 and a 95% confidence interval of 0.1 to 0.9. This suggests that patients receiving HDR brachytherapy had a significantly better survival outcome compared to those who did not receive HDR brachytherapy. A study reported excellent local control and survival rates with a novel low-dose HDR brachytherapy regimen for early-stage endometrial cancer. The 2-year survival rates were significantly high, demonstrating the effectiveness of HDR brachytherapy in early-stage disease [22]. Lee et al. (2013) evaluated clinical outcomes of CT-guided HDR interstitial brachytherapy for primary and recurrent gynecologic cancers. Their findings indicated acceptable local control and survival rates, supporting the use of HDR brachytherapy in various gynecologic cancers [23].

Limitations 

The authors are aware of the study's limitations. Its retrospective nature may introduce biases, and the short median follow-up period of 24 months limits the understanding of long-term outcomes and late toxicities. Most importantly, the absence of randomization restricts causal inferences, highlighting the need for prospective studies and randomized controlled trials to validate these findings comprehensively. Potential selection bias and missing data, particularly for tumor grade and FIGO stage, could affect the results. The variability in HDR brachytherapy protocols and the lack of detailed toxicity and quality-of-life data further limit the findings. 

## Conclusions

In conclusion, HDR brachytherapy appears to be a highly effective treatment for cervical and endometrial cancers, demonstrating significant survival benefits. Continued research and refinement of treatment approaches are essential to maximize its potential and ensure the best outcomes for patients. Future research should focus on prospective studies and randomized controlled trials with longer follow-up periods to validate these findings. Incorporating patient-reported outcomes and detailed toxicity profiles will provide a more comprehensive understanding of HDR brachytherapy's benefits and risks.
